# Multiple states in ongoing neural activity in the rat visual cortex

**DOI:** 10.1371/journal.pone.0256791

**Published:** 2021-08-26

**Authors:** Daichi Konno, Shinji Nishimoto, Takafumi Suzuki, Yuji Ikegaya, Nobuyoshi Matsumoto

**Affiliations:** 1 Graduate School of Pharmaceutical Sciences, The University of Tokyo, Tokyo, Japan; 2 Graduate School of Medicine, The University of Tokyo, Tokyo, Japan; 3 Center for Information and Neural Networks, National Institute of Information and Communications Technology, Suita City, Osaka, Japan; 4 Institute for AI and Beyond, The University of Tokyo, Tokyo, Japan; Georgia State University, UNITED STATES

## Abstract

The brain continuously produces internal activity in the absence of afferently salient sensory input. Spontaneous neural activity is intrinsically defined by circuit structures and associated with the mode of information processing and behavioral responses. However, the spatiotemporal dynamics of spontaneous activity in the visual cortices of behaving animals remain almost elusive. Using a custom-made electrode array, we recorded 32-site electrocorticograms in the primary and secondary visual cortex of freely behaving rats and determined the propagation patterns of spontaneous neural activity. Nonlinear dimensionality reduction and unsupervised clustering revealed multiple discrete states of the activity patterns. The activity remained stable in one state and suddenly jumped to another state. The diversity and dynamics of the internally switching cortical states would imply flexibility of neural responses to various external inputs.

## Introduction

Most neural activity is internally generated without any external sensory input [1]. In terms of electrophysiological metabolism, neural activity at rest (*i*.*e*., when an animal is not engaged in any specific cognitive tasks) continuously consumes as much, or even more, energy than evoked activity [2, 3].

Previous studies demonstrated that this spontaneous (also referred to as internal or ongoing) neural activity in the primary visual cortex (V1) was associated with dynamic activity evoked by external visual stimuli; that is, ongoing activity could encompass and predict evoked activity in V1 [4–7]. These findings suggested that spontaneous activity is not just background noise that perturbs signal processing [8]. Experimental and theoretical studies have shown that ongoing neural activity actively contributes to different functions, including neural development [9, 10], representation of sensory stimuli [4, 11–14], sensory information processing [5, 6, 15–17], intertrial variability in neural and behavioral responses [6, 18–24], memory [25, 26], consciousness [27], and perception [28–30]. However, the internal properties underlying the dynamics of spontaneous neural activity in awake animals remain almost unclear.

We focused on the primary and secondary visual cortices (V1 and V2, respectively) of awake rats as an experimental model of spontaneous cortical activity. To examine whether spontaneous cortical states exist and how dynamic they are, we designed a novel 32-channel electrode array and recorded 32-site electrocorticograms (ECoGs) in V1 and V2 of awake rats while they were presented with a uniform illumination pattern. We then investigated the propagation pattern of ongoing neural activity to explore cortical states. Given the nonlinear properties of extracellular field oscillations, we analyzed cortical activity using nonlinear dimensionality reduction and unsupervised clustering.

## Materials and methods

### Ethical approvals

Animal experiments were performed with the approval of the Animal Experiment Ethics Committee at The University of Tokyo (approval number: P29-7) and according to the University of Tokyo guidelines for the care and use of laboratory animals. These experimental protocols were carried out in accordance with the Fundamental Guidelines for Proper Conduct of Animal Experiment and Related Activities in Academic Research Institutions (Ministry of Education, Culture, Sports, Science and Technology, Notice No. 71 of 2006), the Standards for Breeding and Housing of and Pain Alleviation for Experimental Animals (Ministry of the Environment, Notice No. 88 of 2006) and the Guidelines on the Method of Animal Disposal (Prime Minister’s Office, Notice No. 40 of 1995). All efforts were made to minimize animal suffering.

### Animals

A total of 8 male 8- to 10-week-old Long Evans rats (Japan SLC, Shizuoka, Japan) with a preoperative weight of 300–350 g were individually housed under conditions of controlled temperature and humidity (22 ± 1°C, 55 ± 5%), maintained on a 12:12-h light/dark cycle (lights off from 07:00 to 19:00) with *ad libitum* access to food and water. Rats were habituated to an experimenter by daily handling for 2 days before the experiments.

### Preparation

**Fig 1:**
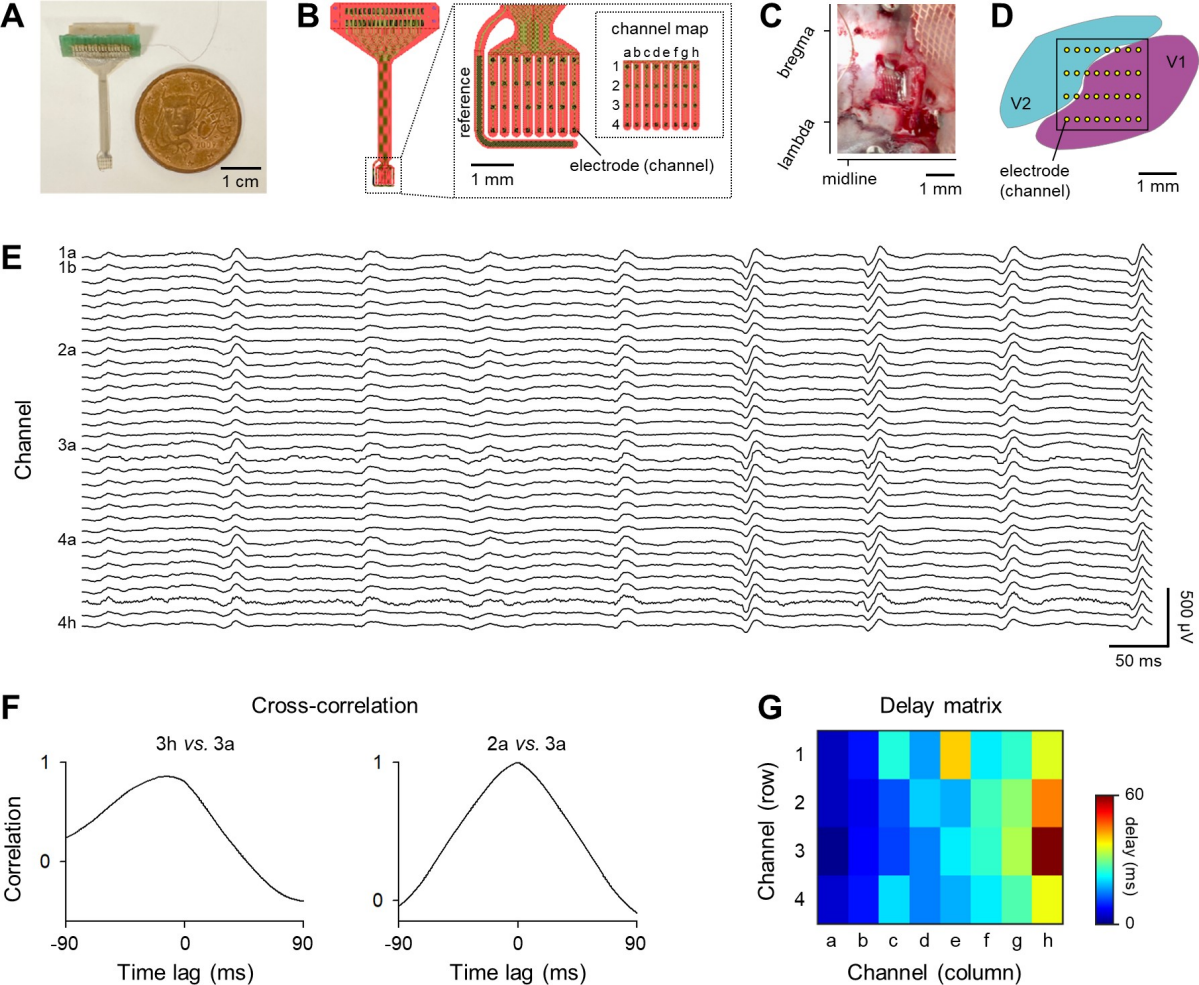
Propagation pattern of spontaneous neural activity in primary and secondary visual cortices recorded by 32-channel ECoG electrodes. ***A***, A photograph of a custom-made 32-channel ECoG electrode array. ***B***, A graphic diagram of the ECoG electrode array shown in ***A***. A number and a letter were allocated to each channel to identify a row and a column, respectively. ***C***, A photograph of a surgical window. The ECoG electrode array was placed on the cortical surface in the cranial window. ***D***, The electrode array covered the surface of the primary and secondary visual cortices (V1 and V2, respectively). ***E***, Representative traces of 0.9-s ECoG signals. Note that we defined a block of 0.9-s 32-channel ECoGs as an episode. ***F***, *Left*: Representative cross-correlation function between ECoGs (shown in ***E***) recorded by the latest (*e*.*g*., 3h) and the reference (*e*.*g*., 3a) channels. *Right*: The same as *left*, but between the earliest (*e*.*g*., 2a) and the reference channels. ***G***, A representative pseudocolored matrix of delay between ECoGs (shown in ***E***) recorded by the reference channel (*e*.*g*., 3h) and the other channels. Hot and cold colors indicate long and short delays, respectively.

We designed and fabricated a parylene-C[poly(chloro-paraxylylene)]-based 32-channel flexible high-density electrode array plus one reference electrode (Fig 1A and 1B) [31, 32]. The electrode array was separated into eight strips, each of which consisted of four individual platinum electrode channels. The interelectrode distance (between centers) was 700 μm along the stripe and 350 μm perpendicular to the stripe. Each electrode channel was a square 100 μm on a side, an area of which was approximately 0.01 mm^2^. The whole area (2.5 mm × 2.5 mm) of the array was set such that it would sufficiently cover V1 and V2. The thickness of the array was 20 μm. The electrode impedance was 17.9 ± 6.3 kΩ (tested at 1 kHz, 10 nA, 300 cycles).

### Surgery

General anesthesia for rats was induced and maintained by 2–3% and 1–2% isoflurane gas in air, respectively, with careful inspection of the animal condition during the whole surgical procedure. Veterinary ointment was applied to the rats’ eyes to prevent drying. The skin was sterilized with povidone iodine and 70% ethanol wherever we made an incision.

After sufficient anesthesia, we mounted an animal onto a stereotaxic apparatus with two ear bars and a nose clamp (SR-6R-HT; Narishige, Tokyo, Japan) according to general surgical procedures [33]. An incision was made from the area between the eyes to the back of the head. Circular craniotomies (approximately 0.9 mm in diameter) were made using a high-speed drill (SD-102; Narishige). We then performed a 4.0-mm square craniotomy centered at 4.5 mm posterior and 3.5 mm lateral to bregma so that the craniotomy would cover both V1 (5.5 mm posterior and 4.0 mm lateral to bregma) and V2 (medial V2 (V2M); 5.0 mm anterior and 2.5 mm lateral to bregma) (Fig 1C and 1D). The electrode array was placed into the square cranial window, either epidurally or subdurally, on the cortical surface to record ECoGs. An additional stainless-steel screw was implanted in the bone above the cerebellum (9.6 mm anterior and 1.0 mm bilateral to bregma) as a ground electrode. The whole electrode assembly was secured to the skull using stainless-steel screws and dental cement.

After all surgical procedures, anesthesia was terminated. Each animal was allowed to spontaneously wake up from anesthesia. Following surgery, each animal was individually housed in a transparent Plexiglas cage with free access to water and food. We carefully checked the postoperative condition of the animals. The animals were habituated to an experimenter again by handling and to a recording apparatus for a couple of days until the experiment. After at least 5 days of recovery from surgery, we commenced electrophysiological recordings.

While our experimental protocols have a mandate to humanely kill animals if they exhibit any signs of pain, prominent lethargy and discomfort, we did not observe such symptoms in any rats used in this study.

### Apparatus

We used a small open field with transparent walls located in a dark, soundproof room. The field measured 35 cm in width, 46 cm in depth, and 18 cm in height and was surrounded by four monitors. Uniform gray images (blank stimuli) and monochrome black-and-white stripe images (grating stimuli) on the monitors were extended using two mirrors on the top and bottom of the field.

### *In vivo* electrophysiology

We placed each rat in the field to freely explore for 10 minutes and connected the electrode assembly to a digital low-noise 32-channel amplifier (CerePlex M; Blackrock, UT, USA). The multiplexed output of the headstage was conducted through lightweight multiwired tethers and a commutator to a multichannel data acquisition system (Cerebus; Blackrock). We recorded ECoG signals for 4 h while visual stimuli were presented to an awake rat. Neural activity in V1 and V2 was continuously recorded while the rat was presented with visual stimuli that were alternatively switched and composed of 1-s grating stimuli and 2.5-s blank stimuli. We extracted the center of the blank stimuli (0.9-s for duration, 3.5-s for interstimulus interval) and analyzed the individually separated neural activity (see the next section). For all recordings, electrophysiological signals were amplified and digitized at 2 kHz and filtered between 0.1 Hz and 500 Hz.

### Data analysis

All data analyses were performed using custom-made MATLAB 2020a (MathWorks, MA, USA) and Python 3 routines. Summarized data are reported as the mean ± the standard error of the mean (SEM). The null hypothesis was statistically rejected when *P* < 0.05 unless otherwise specified. When multiple comparisons were required, we corrected the original significance level (*i*.*e*., 0.05) with Bonferroni’s correction and compared the original *P* values with the corrected significance level.

We extracted 0.9-s ECoG data approximately every 3.5 s and obtained approximately 4,000 segments per animal (see the previous section; Fig 1E).

We performed the following analyses for each segment (referred to as ‘an episode’, hereafter); note that an episode was composed of 0.9-s ECoGs from 32 channels. For a pair of given two electrode channels during an episode, we (circularly) shifted two ECoG signals to calculate its cross-correlation function *C*(τ) (-90 ms < τ < 90 ms) between the signals (Fig 1F) [34]. When *C*(τ_max_) took the maximum value, we defined a time lag as the amount of time shift (*i*.*e*., τ_max_). We repeated this calculation for all 496 (*i*.*e*., _32_C_2_) possible pairs of ECoG signals. For each ECoG channel (among the 32), we counted the number of ‘earlier’ channels (ranging between 0 and 31) than itself based on the time lag. We defined the most ‘preceding’ channel as a channel that had either no or fewest ‘earlier’ channels. We computed the time lag of each channel relative to the most ‘preceding’ channel and created a pseudocolored 32-element matrix (named a ‘delay matrix’) based on the time lags (Fig 1G); note that a ‘delay’ for a given channel *j* was defined as the sum of time lags between channel *j* and the other channels. We then repeated the same calculation for all episodes; that is, we produced as many delay matrices as episodes.

**Fig 2:**
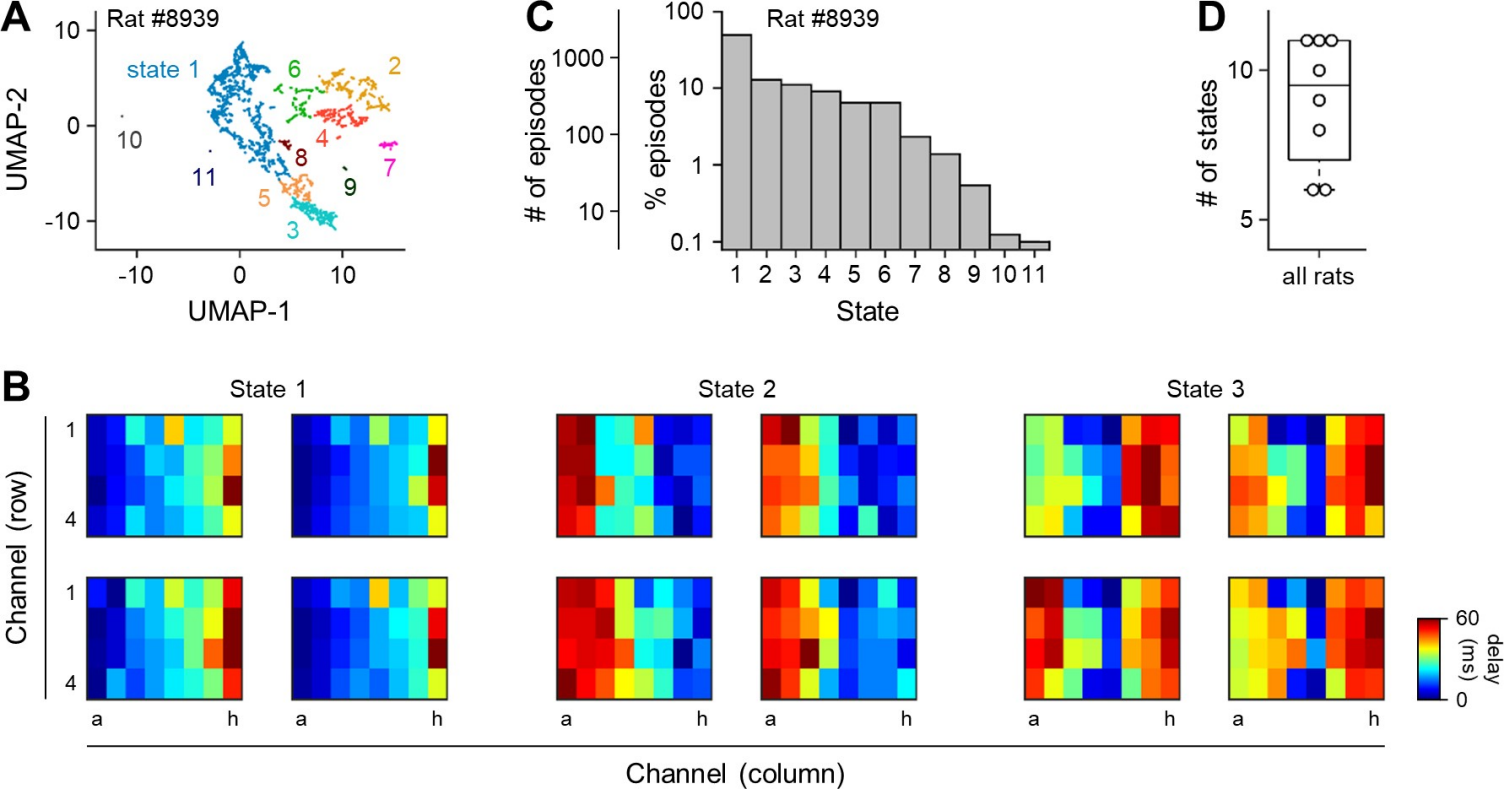
Diversity in the propagation patterns of neural activity in the visual cortices. ***A***, A representative map of ECoG episodes. Each of them was embedded in a two-dimensional UMAP space. All episodes were grouped by the RCC routines to identify states. ***B***, Representative delay matrices for states 1 (*left*), 2 (*middle*), and 3 (*right*) shown in ***A***. ***C***, The percentage and the number of episodes classified into a given state shown in ***A***. ***D***, The number of states of propagation patterns for all rats tested in this study. *Abbreviations*: UMAP, uniform manifold approximation and projection; RCC, robust continuous clustering.

To better understand the transition in neural activity states, we used two algorithms (*i*.*e*., nonlinear dimensionality reduction and unsupervised clustering). For nonlinear dimensionality reduction, we utilized uniform manifold approximation and projection (UMAP) (Fig 2A) [35, 36]. After we removed jamming noises (*e*.*g*., hum noises and muscular twitches) from the signal, we applied UMAP to reduce 32 dimensions of each delay matrix into a point in a two-dimensional space (Python implemented with the following parameters: *n_neighbors* = 3, *min_dist* = 0.1, *n_components* = 2, and *metric* = ‘euclidean’). We repeated the same procedure for all delay matrices and used a robust continuous clustering (RCC) algorithm to detect states in the UMAP space (Python implemented with the following parameters: *clustering_threshold* = 100, *k* = 100, and *measure* = ‘euclidean’) (Fig 2A–2C, S1 Fig) [37], which was subsequently repeated for all rats (Fig 2D). The number of states was automatically determined and denoted by *K* hereafter.

**Fig 3:**
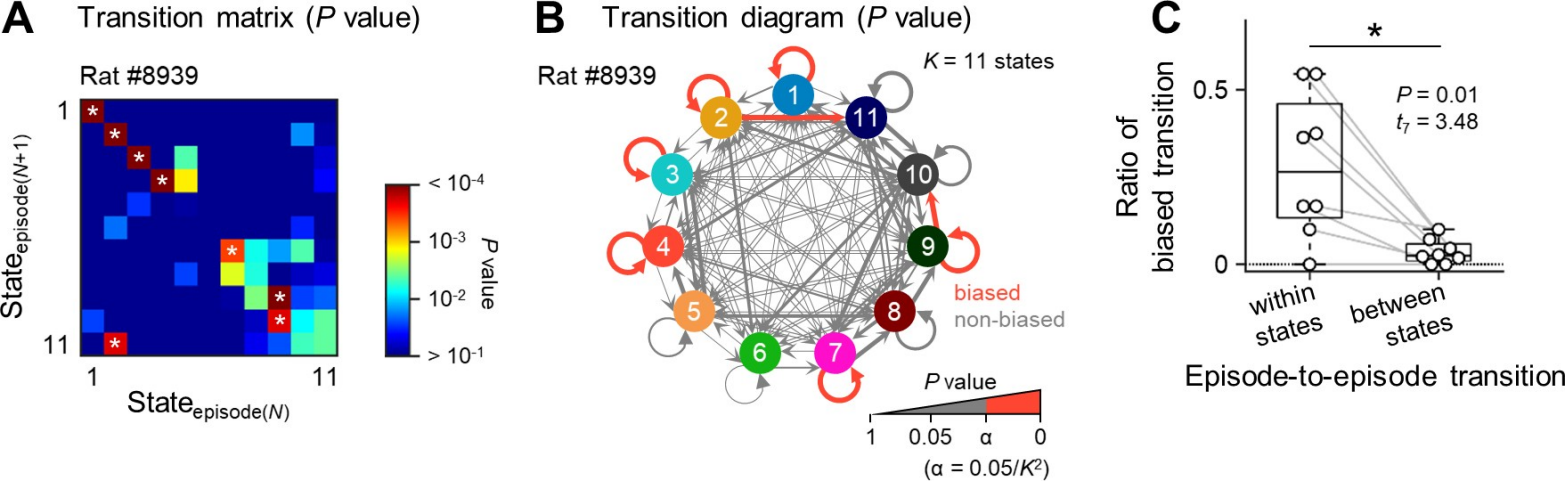
Dynamics of transition patterns of neural activity. ***A***, Transition from the *N*^th^ episode (*i*.*e*., episode(*N*)) to the next was transformed into a state-to-state transition matrix, where a state to which the episode(*N*) belonged was represented as a state_episode(*N*)_. Warmer colors indicate that a given state-to-state transition was significant. Note that *white* asterisks in the heat map indicate *P* < 0.05/121 and that these transitions were named biased transitions. ***B***, The transition matrix (shown in ***A***) was transformed into a transition diagram. *Red* and *gray* arrows indicate biased and nonbiased state-to-state transitions, respectively. Thicker arrows represent lower *P* values. ***C***, The ratio of biased transitions to all transitions within the identical states was significantly higher than that between distinct states.

*K* states could produce *K*^2^ transition patterns between episodes. The transition from the *N*^th^ episode (*i*.*e*., episode(*N*)) to the next was transformed into a state-to-state transition matrix, where a state to which the episode(*N*) belonged was represented as a ‘state_episode(*N*)_’. For each transition pattern, we counted the number of surrogate data points that had more transitions than the real data and divided the number plus 1 by 10,001 to estimate a *P* value for the real data. All *P* values were summarized and depicted in a pseudocolored *K×K* ‘transition matrix’ (Fig 3A) and a ‘transition diagram’ (Fig 3B). If the *P* value for a given transition pattern was less than the *post hoc* corrected significance level (*i*.*e*., 0.05/*K*^2^), it was considered a biased transition pattern. We then classified *K*^2^ transition patterns into *K* ‘within-states’ (*i*.*e*., episode-to-episode transitions within identical states) and *K*^2^-*K* ‘between-states’ (*i*.*e*., transitions between distinct states). For both ‘within-states’ and ‘between-states’, we calculated the ratio of the biased transition patterns (Fig 3C).

## Results

Using a mesh-like planar electrode array (Fig 1A and 1B), we recorded ECoGs from 32 sites in the V1 and V2 of unrestrained rats in an open field (Fig 1C and 1D) [31, 32]. We continuously monitored spontaneous neural activity for several hours and extracted 0.9-s ECoG data segments every 3.5 s. Each time segment was referred to as an ‘episode’, hereafter (Fig 1E). Based on the peak offset (*e*.*g*., time lag in propagation) in the cross-correlation function of all possible pairs of ECoG electrode channels (Fig 1F), we created a delay matrix for an episode (Fig 1G). We regarded this delay matrix as an index of spontaneous neural activity.

For a given animal, we created as many delay matrices as episodes. We then addressed the question of how many spontaneous activity states emerged in the cortices based on visual attributes. We reduced 32 dimensions of the delay matrices to two dimensions using UMAP [35] and classified the matrices into several states using RCC, an unsupervised clustering method [37]; note that the number of states was not predetermined but was automatically determined in the RCC method (Fig 2A and 2B). These analyses indicated that the activity patterns were discrete and subclustered in the state space. To address the question of whether the visual cortical activity patterns emerged uniformly or more preferentially and specific to certain states, we calculated the number and percentage of episodes in each state. The episodes were not evenly distributed in a specific state, and most of them were biased in specific states (Fig 2C). Among all animals tested, the number of states ranged between 6 and 11 (Fig 2D), and similar biased distributions of episodes were found in all animals (S1 Fig).

We next investigated the time-series dynamics of the propagation patterns by characterizing whether and how the propagation pattern remained temporally stable or dynamically switched to another. To issue this question, we visualized the state-to-state dynamics by calculating the probability of episode-to-episode transitions (Fig 3A and 3B). The transitions also exhibited ‘biased’ patterns; that is, some specific state transitions occurred more frequently than a chance level. For example, we found that, for the rat #8939, the transition from the state 2 to 11 significantly occurred compared with the chance level, whereas the occurrence of the transition from state 2 to 10 was not significantly different from the chance level (Fig 3A). For all rats used in this study, the number of state-to-state transitions for all of two sequential (*i*.*e*., the *N*^th^ to the next) episodes was biased for the real data but not for the surrogate (S2 Fig). The ratio of biased transitions within the identical states was significantly larger than that between distinct states (Fig 3C; *P* = 0.01, *t*_7_ = 3.48, *n* = 8 rats, paired *t*-test). These results suggested that the cortical activity propagation pattern was temporally static, unlike Brownian motion or random walks.

## Discussion

Using our new custom-made flexible ECoG electrode array, we simultaneously recorded spontaneous neural activity from V1 and V2 of unrestrained rats and quantified the propagation patterns of neural activity using time-lag matrices. Based on nonlinear dimensionality reduction and unsupervised clustering, we further characterized the internal states of spontaneous neural activity and revealed that the transition of propagation patterns occurred more frequently within states than between different states.

To monitor the population activity in the visual cortex of awake rats with a fine resolution in space and time, we designed and fabricated a 32-channel high-density ECoG electrode array. The ECoG electrode array used in this study has much smaller electrode channels and captures neural activity in higher spatial resolution than previous ECoG arrays used mostly for humans [32, 38–41]. The ECoG recording method generally has some advantages compared with other *in vivo* extracellular/intracellular electrophysiological recordings. Extracellular single-unit or multiunit recordings observe suprathreshold firing activity at the single-cell level [42, 43], whereas ECoG recording allows for less invasive monitoring of summed synaptic activity from multiple (~ 10^5^ in humans, for example [44]) neurons [45]. More strictly, intracellular recording techniques measure the synaptic activity of individual neurons; however, simultaneous *in vivo* intracellular recordings from large populations of neurons are still technically difficult. Consequently, our recording method allows for recording collective synaptic activity less invasively on a fine temporal scale for a long time.

For the current analysis, we used nonlinear methods as well as linear methods. Analyses of extracellular oscillations have often focused on their frequencies, such as delta, beta, theta, and gamma; however, multichannel ECoG signals are spatiotemporally complex. We thus combined linear and nonlinear methods for the data analysis. Moreover, in UMAP, the larger *n_neighbors* value generally results in the even smaller number of clusters, as is consistently seen in our analysis. Using the delay matrices, we intended to preserve as local structure as possible to determine cortical states and set 3 as *n_neighbors*; note that, strictly speaking, it is hard to determine the truly appropriate value of *n_neighbors* because UMAP works in a topological, not metric space.

Spontaneous neural activity in the visual cortex of anesthetized cats could be divided into more than 30 states using voltage-sensitive dye imaging [4]. Voltage-sensitive dye imaging can measure synaptic membrane potentials. In this sense, the imaging technique reliably captured visual cortical neural states based on a large population of neurons. However, imaging methods were different from electrophysiology in terms of temporal resolution. Electrophysiological recording generally enables us to capture fast kinetics of neural signals more precisely than imaging methods. Thus, our analysis might have emphasized fast fluctuations in neural signals.

Moreover, we took into account the propagation patterns of neural activity not only in V1 but also in V2 for analysis. Although V2 is unique in the function compared to the V1 [46], we mixed the electrophysiological signals in V1 and V2 together for analysis, because we intended to investigate interregional (*i*.*e*., V1 and V2) propagation of the neural signals. Secondary sensory (*e*.*g*., visual, auditory, and somatosensory) cortical neurons are generally innervated by neurons in the primary sensory cortex [47]. Specifically, corticocortical projections from V1 to V2 (medial V2 (V2M)) have been reported, and *vice versa* [48]. Nevertheless, how neural signals are propagated over the two regions is poorly understood. Thus, we collectively, not separately, analyzed the data of V1 and V2.

Spontaneous neural activity encompasses the stability and switching of cortical states. Apparently, state-to-state transitions seem to be a random jump caused by a high level of noises because we did not present rats with any specific visual stimuli [8, 49, 50]. Although we cannot exclude the possibility, we have come up with other plausible mechanisms for the state transitions in the visual cortex: (i) neuromodulatory signals and (ii) vestibular signals. First, we consider that such visual cortical state dynamics (*i*.*e*., stability and switching) is associated with neuromodulatory functions such as noradrenergic and cholinergic neural activities. The locus coeruleus neurons innervate V1, modulate V1 activity by releasing noradrenaline [51], and exhibit tonic and phasic activity [52]. Since increased tonic (baseline) activity in the locus coeruleus is linked with less effective engagement in behavioral tasks [52], the tonic activity may dominate the locus coeruleus when the rat is sedated during our task. Thus, such robust activity in the locus coeruleus may gradually evolve visual cortical neural activity in a fluid, not abrupt manner. Furthermore, another possible mechanism underlying the evolution of the cortical state dynamics is intermittent and sustained modulation of cortical activity associated with rapid and long-lasting dilations of the pupil, respectively [53, 54], although we cannot completely exclude the possibility that the distinct patterns of neural activity might stem from the discrete periods of time during recording. Pupil size is assumed to be reliably indicative of neural activity in the locus coeruleus, a principal brain region for synthesis of noradrenaline. In addition to noradrenaline, the release of acetylcholine strongly controls cortical states [53–55]. A recent study demonstrated that the rapid dilations of the pupil were tracked by the phasic noradrenergic activity, whereas the sustained changes in pupil diameter (*e*.*g*., during locomotion) were associated with the tonic cholinergic activation [53]. These multiple neuromodulatory transmitter systems could exert an influence on the cortical state dynamics and trigger the state transition in behaving rats. Moreover, in the current study, rats were freely behaving and thus ascending vestibular signals were incessantly transmitted to various [56]. Not only the neuromodulators but also the vestibular signals may contribute to the visual cortical activity. Still, how the states determined by extracellular oscillations are correlated with visual functions such as orientation and direction selectivity has yet to be investigated [57]. We assume that the variety in spontaneously switching cortical dynamics is determined by extra and intracortical connectivity and variable synaptic inputs. The ongoing cortical activity may be associated with intrinsic default states to prepare flexible responses to various sensory stimuli in line with the winnerless competition dynamics [58] and the temporal patterns of synchronized neural activity [59, 60].

## Supporting information

S1 Fig The percentage of episodes in each state for all rats. Fraction of episodes classified into each state for rats named #8907, #8908, #8909, #8934, #8939, #9041, #9589, and #9640.
(PDF)

S2 Fig The number of state-to-state transitions for all of two sequential episodes for all rats. The number of transitions from the *N*^th^ episode (*i*.*e*., episode(*N*)) to the next was transformed into a state-to-state matrix, where a state to which the episode(*N*) belonged was represented as a state_episode(*N*)_. For each animal, episodes in each cluster were sampled with replacement, which generated a random sequence of episodes. This process was repeated 10,000 times. The randomization produced 10,000 matrices, each cell of which contained the number of the transitions. The average of the matrices was displayed as the surrogate. ***A-H***, Matrices for the real (*top*) and the surrogate (*bottom*) data for the rats named #8907, #8908, #8909, #8934, #8939, #9041, #9589, and #9640, respectively.
(PDF)
