# Supplementary material for: Multiple states in ongoing neural activity in the rat visual cortex
Source: PLoS One. 2021 Aug 26;16(8):e0256791. doi: 10.1371/journal.pone.0256791 (PMC8389421; doi:10.1371/journal.pone.0256791)
Supplement: S1 Fig — Fraction of episodes classified into each state for rats named #8907, #8908, #8909, #8934, #8939, #9041, #9589, and #9640. (PDF) [file pone.0256791.s001.pdf]

Konno *et al.*, Supporting Information

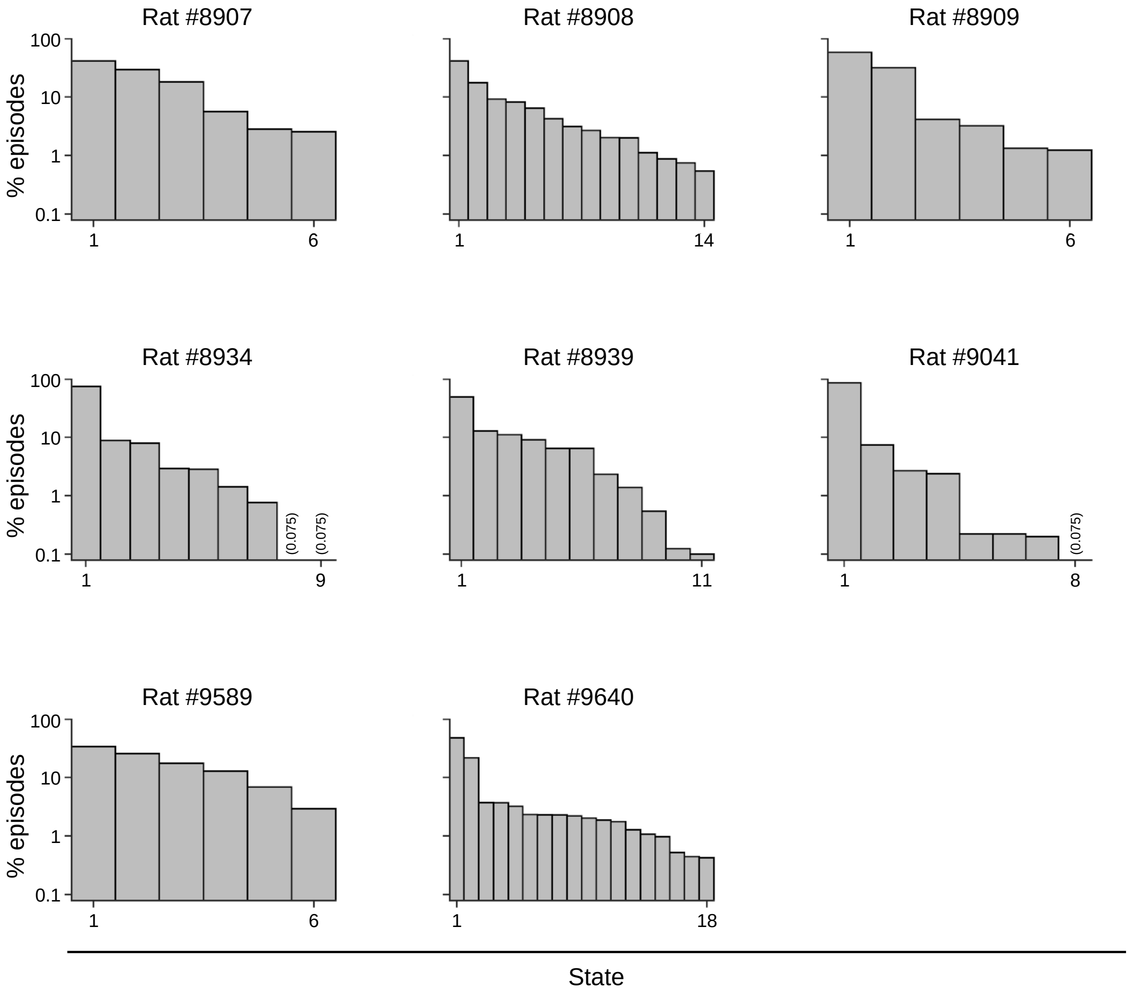

S1 Fig (related to Fig 2C) | The percentage of episodes in each state for all rats. Fraction of episodes classified into each state for rats named #8907, #8908, #8909, #8934, #8939, #9041, #9589, and #9640.
