# Supplementary material for: Multiple states in ongoing neural activity in the rat visual cortex
Source: PLoS One. 2021 Aug 26;16(8):e0256791. doi: 10.1371/journal.pone.0256791 (PMC8389421; doi:10.1371/journal.pone.0256791)
Supplement: S2 Fig — The number of transitions from the Nth episode (i.e., episode(N)) to the next was transformed into a state-to-state matrix, where a state to which the episode(N) belonged was represented as a stateepisode(N). For each animal, episodes in each cluster were sampled with replacement, which generated a random sequence of episodes. This process was repeated 10,000 times. The randomization produced 10,000 matrices, each cell of which contained the number of the transitions. The average of the matrices was displayed as the surrogate. A-H, Matrices for the real (top) and the surrogate (bottom) data for the rats named #8907, #8908, #8909, #8934, #8939, #9041, #9589, and #9640, respectively. (PDF) [file pone.0256791.s002.pdf]

Konno *et al.*, Supporting Information

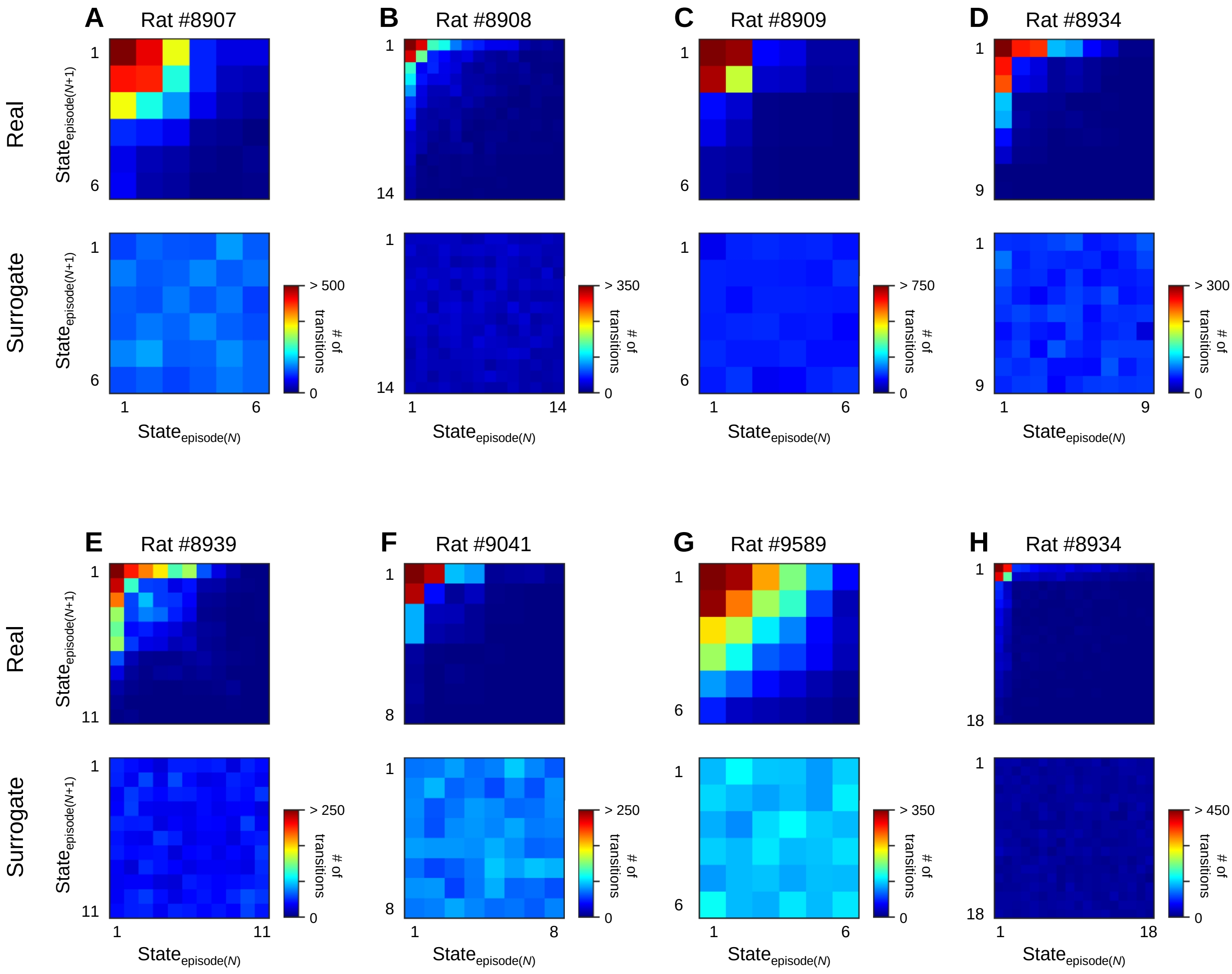

S2 Fig (related Fig 3A) | The number of state-to-state transitions for all of two sequential episodes for all rats.

The number of transitions from the Nth episode (*i.e.*, episode(N)) to the next was transformed into a state-to-state matrix, where a state to which the episode(N) belonged was represented as a state$_{episode(N)}$. For each animal, episodes in each cluster were sampled with replacement, which generated a random sequence of episodes. This process was repeated 10,000 times. The randomization produced 10,000 matrices, each cell of which contained the number of the transitions. The average of the matrices was displayed as the surrogate. ***A-H***, Matrices for the real (*top*) and the surrogate (*bottom*) data for the rats named #8907, #8908, #8909, #8934, #8939, #9041, #9589, and #9640, respectively.
